# Development and performance assessment of a qualitative SYBR® green real-time PCR assay for the detection of *Aspergillus versicolor* in indoor air

**DOI:** 10.1007/s00253-015-6785-9

**Published:** 2015-07-17

**Authors:** X. Libert, C. Chasseur, S. Bladt, A. Packeu, F. Bureau, N. H. Roosens, S. C. J. De Keersmaecker

**Affiliations:** Platform Biotechnology and Molecular Biology, Scientific Institute of Public Health (WIV-ISP), J. Wytsmanstraat 14, 1050 Brussels, Belgium; Health and Environment, Scientific Institute of Public Health (WIV-ISP), J. Wytsmanstraat 14, 1050 Brussels, Belgium; Cellule Régionale d’Intervention en Pollution Intérieure (CRIPI), Brussels Environment (IBGE), Avenue du Port 86C/3000, 1000 Bruxelles, Belgium; Mycology and Aerobiology, Scientific Institute of Public Health (WIV-ISP), J. Wytsmanstraat 14, 1050 Brussels, Belgium; Cellular and Molecular Immunology, Groupe Interdisciplinaire de Génoprotéomique Appliquée (GIGA), Université de Liège (ULg), Avenue de l’Hôpital, 1 (B34), 4000 Sart-Tilman, Belgium

**Keywords:** *Aspergillus versicolor*, Indoor air, Public health, Real-time PCR, SYBR® green, Performance assessment

## Abstract

Currently, contamination of indoor environment by fungi and molds is considered as a public health problem. The monitoring of indoor airborne fungal contamination is a common tool to help understanding the link between fungi in houses and respiratory problems. Classical analytical monitoring methods, based on cultivation and microscopic identification, depend on the growth of the fungi. Consequently, they are biased by difficulties to grow some species on certain culture media and under certain conditions or by noncultivable or dead fungi that can consequently not be identified. However, they could have an impact on human health as they might be allergenic. Since molecular methods do not require a culture step, they seem an excellent alternative for the monitoring of indoor fungal contaminations. As a case study, we developed a SYBR® green real-time PCR-based assay for the specific detection and identification of *Aspergillus versicolor*, which is frequently observed in indoor environment and known to be allergenic. The developed primers amplify a short region of the internal transcribed spacer 1 from the 18S ribosomal DNA complex. Subsequently, the performance of this quantitative polymerase chain reaction (qPCR) method was assessed using specific criteria, including an evaluation of the selectivity, PCR efficiency, dynamic range, and repeatability. The limit of detection was determined to be 1 or 2 copies of genomic DNA of *A. versicolor*. In order to demonstrate that this SYBR® green qPCR assay is a valuable alternative for monitoring indoor fungal contamination with *A. versicolor*, environmental samples collected in contaminated houses were analyzed and the results were compared to the ones obtained with the traditional methods.

## Introduction

The effects of allergenic molds on public health are well documented (Bellanger et al. 2009; Packeu et al. 2012; Reboux et al. 2010). Indeed, molds can produce mycotoxins, spores, hyphae, and wall fragments containing (1 → 3)-β-D-glucans and proteins which could induce allergic reactions of types I, III, and IV (Douwes et al. 2003; Seo et al. 2008). Some studies revealed an association between the fungal levels in the air and the occurrence of allergy (Horner et al. 1995; Meheust et al. 2014; Mendell et al. 2011; Reboux et al. 2010) and between water-damaged buildings and human exposure to fungal contamination present in indoor air (Jones et al. 2011; Vesper et al. 2013). Presently, with the climate change, the new energy conservation measures, the development of the urbanization, and the considerable amount of time spent inside buildings, people are increasingly exposed and might be more susceptible to developing a respiratory problem caused by a fungal contamination (de Ana et al. 2006; Mendell et al. 2011; Sharpe et al. 2014). More specifically, *Aspergillus versicolor* is one of the most important fungal contaminants of houses (Beguin and Nolard 1994; Packeu et al. 2012), and this species is known to produce allergenic compounds (Benndorf et al. 2008), which can have an implication in the development of asthma (de Ana et al. 2006).

Usually in routine analysis, the detection and identification of indoor airborne fungi are based on culture, microscopic visualization, and visual counts. However, this classical approach requires an important level of expertise and is time-consuming (Vesper 2011). Another drawback of this approach is that it depends on the growth of the culture, which is known to be affected by the growth media chosen, the culture conditions (especially temperature and humidity), the incubation time (some species grow faster than others), or competition between species. Furthermore, a part of the sampled biological fraction is dead and noncultivable and therefore is not always detected by traditional analysis methods, although it could have an impact on human health as it might be allergenic. Therefore, this could lead to an underestimation of the total fungal airborne community and level of the contamination classically determined by colony-forming units (CFU) on an agar plate (Pitkaranta et al. 2011; Vesper 2011).

Molecular techniques like the real-time quantitative polymerase chain reaction (qPCR), amplifying a specific DNA sequence in the fungal genome with a real-time detection of the amplification products, have been proposed as an alternative to these classical detection methods (Black et al. 2013; Haugland et al. 2004; Timothy et al. 2004). Besides being fast, sensitive, and specific, qPCR also gives the advantage of being independent from culture.

Most often qPCR methods designed for fungal detection are based on the ribosomal gene complex and particularly on its internal transcribed spacers (ITS) (Bellanger et al. 2009; Chemidlin Prevost-Boure et al. 2011; Costa et al. 2001; Johnson et al. 2012; Melkin et al. 2004; Michealsen et al. 2006; Roussel et al. 2013), which are noncoding regions of the fungal rDNA (ITS 1 and ITS 2) flanked by the small subunit (SSU) rRNA and by the large subunit (LSU) rRNA genes. The sequence variation of ITS regions has led to their use in phylogenetic studies of many different organisms (Nilsson et al. 2008). These ITS regions are selected for qPCR assays due to their good conservation and their weak level of polymorphism amongst DNA sequences of the same genus (Chemidlin Prevost-Boure et al. 2011; Costa et al. 2001; Iwen et al. 2002). Therefore, they offer the possibility to develop specific molecular methods for detection at the genus or even at the species level (Hinrikson et al. 2005; Nilsson et al. 2008; Schoch et al. 2012).

The qPCR methods are started to be more frequently used for environmental investigations and monitoring of the most common fungal contaminants present inside buildings (Bellanger et al. 2009; Roussel et al. 2013), such as performed by the US Environmental Protection Agency (EPA). Indeed, the EPA has developed a large set of qPCR methods based on the TaqMan® chemistry, as real-time detection system of the amplified products aimed for indoor fungi monitoring (*A. versicolor* included) (Haugland et al. 2004; United States Environmental Protection Agency 2014). These labeled probe-based qPCR assays are highly specific. An alternative to TaqMan® method is the SYBR® green chemistry. Here, the detection of amplification is based on an intercalating fluorescent dye, independent of the use of a labeled probe. The specificity of this method is determined by the primers’ specificity and by the melting temperature (*T*_m_) of the amplicon. This approach is also sensitive and fast but cheaper than the probe-based one. However, despite these advantages, not many SYBR® green assays exist yet for the detection and identification of airborne indoor molds. To the best of our knowledge, no SYBR® green based detection and identification method for *A. versicolor* has been reported yet.

Additionally, currently, even if many qPCR methods exist for the fungal detection, no specific guidelines are proposed concerning the assessment of their performance. This is in contrast to other domains where real-time PCR methods are already used during many years for the detection and identification of specific targets, such as for the detection of genetically modified organisms (GMO) in food and feed (Broeders et al. 2014; European Network of GMO Laboratories (ENGL) 2008; ENGL 2015). Recently, the guidelines established for the validation of real-time PCR methods for GMO detection have been used to evaluate qPCR assays for the detection and identification of bacterial pathogens (Barbau-Piednoir et al. 2013b). However, for fungal qPCR detection methods, this has not yet been done.

The present study reports on the development of a SYBR® green qPCR method for *A. versicolor* detection. The primers designed in this study are selected in the ITS region. The performance of the developed qPCR assay was subsequently assessed, using the guidelines for validation of qualitative real-time PCR methods based on criteria defined for GMO. The PCR efficiency, dynamic range, sensitivity, selectivity, and repeatability of the developed *A. versicolor* SYBR® green qPCR were evaluated and discussed. Finally, a proof of concept for the developed qPCR method was delivered using air samples collected in two contaminated houses, by comparing the qPCR results to the results obtained with traditional analysis.

## Materials and methods

### Fungal strains

All the fungal species (*A. versicolor*, *Aspergillus creber*, *Aspergillus sydowii*, *Aspergillus fumigatus*, *Alternaria alternata*, *Cladosporium cladosporoïdes*, *Cladosporium herbarum*, *Cladosporium sphaerospermum*, *Penicillium chrysogenum*, *Stachybotrys charatum*, *Ulocladium botrytis*) and strains used in this study are listed in Table 1. All of them were purchased from the BCCM/IHEM collection (Scientific Institute of Public Health in Brussels, Belgium).

**Table 1:** Selectivity evaluation of SYBR® green qPCR *Aversi*_*ITS* assay

Genus	Species	Reference BCCM/IHEM^a^	Positive signal	*C*_q_ Mean ± SD	*T*_m_ Mean ± SD (°C)
***Aspergillus***	***versicolor***	**IHEM 18884**	**Yes**	**26.13 ± 0.06**	**76.25 ± 0.35**
*Aspergillus*	*versicolor*	IHEM 1323	Yes	26.51 ± 0.67	76.50 ± 0.00
*Aspergillus*	*versicolor*	IHEM 1355	Yes	28.67 ± 0.23	76.38 ± 0.25
*Aspergillus*	*versicolor*	IHEM 2023	Yes	26.73 ± 0.06	76.25 ± 0.35
*Aspergillus*	*versicolor*	IHEM 2157	Yes	27.2 ± 0.11	76.50 ± 0.00
*Aspergillus*	*versicolor*	IHEM 2788	Yes	26.02 ± 0.27	76.50 ± 0.00
*Aspergillus*	*versicolor*	IHEM 29832	Yes	28.18 ± 0.16	76.50 ± 0.00
*Aspergillus*	*versicolor*	IHEM 6598	Yes	26.54 ± 0.59	76.25 ± 0.29
*Aspergillus*	*versicolor*	IHEM 9674	Yes	26.30 ± 1.39	76.63 ± 0.25
*Aspergillus*	*versicolor*	IHEM 10351	Yes	26.74 ± 0.08	76.25 ± 0.35
*Aspergillus*	*versicolor*	IHEM 19014	Yes	24.26 ± 0.44	76.75 ± 0.29
*Aspergillus*	*versicolor*	IHEM 19210	Yes	26.24 ± 0.53	76.75 ± 0.29
*Aspergillus*	*versicolor*	IHEM 19256	Yes	27.45 ± 0.87	76.63 ± 0.25
*Aspergillus*	*versicolor*	IHEM 22014	Yes	28.10 ± 0.10	76.50 ± 0.00
*Aspergillus*	*versicolor*	IHEM 22975	Yes	26.54 ± 0.07	76.50 ± 0.00
*Aspergillus*	*versicolor*	IHEM 24424	Yes	28.05 ± 0.99	76.25± 0.29
*Aspergillus*	*creber*	IHEM 2646	Yes	30.70 ± 0.70	76.50 ± 0.00
*Aspergillus*	*sydowii*	IHEM 895	Yes	30.18 ± 0.13	76.25 ± 0.35
*Aspergillus*	*sydowii*	IHEM 1360	Yes	37.34 ± 1.84	76.25 ± 0.35
*Aspergillus*	*sydowii*	IHEM 20347	Yes	33.34 ± 0.88	76.50 ± 0.00
*Aspergillus*	*fumigatus*	IHEM 1365	No	/	/
*Aspergillus*	*fumigatus*	IHEM 3562	No	/	/
*Alternaria*	*alternata*	IHEM 4969	No	/	/
*Cladosporium*	*cladosporoïdes*	IHEM 0859	No	/	/
*Cladosporium*	*herbarum*	IHEM 2268	No	/	/
*Cladosporium*	*sphaerospermum*	IHEM 1011	No	/	/
*Penicillium*	*chrysogenum*	IHEM 4151	No	/	
*Penicillium*	*chrysogenum*	IHEM 20859	No	/	/
*Stachybotrys*	*charatum*	IHEM 0359	No	/	/
*Ulocladium*	*botrytis*	IHEM 0328	No	/	/

*yes* defined as a positive signal i.e. amplification with a *C*_q_ ≤ 40, and *T*_m_ value (°C) as expected; *no* defined as no amplification. *C*_q_ mean ± SD and *T*_m_ mean ± SD were based on two runs per extract from two independent DNA extracts for each strain which has given a positive signal in qPCR using 1000 theoretical genomic copies. The strain in bold is the reference used for the performance assessment and was fully characterized as *A. versicolor*. The BCCM/IHEM 18884 strain was collected and purified from a contaminated house by CRIPI and is used as a reference strain for allergy studies by the CRIPI

^a^IHEM/BCCM collection, Mycology and Aerobiology, Scientific Institute for Public Health, rue Juliette Wytsman 14, 1050 Brussels, Belgium

### Culture conditions and DNA extraction

The fungal strains were grown in a S10 Sabouraud liquid medium (Bio-Rad, Temse, Belgium) at 25 °C with constant agitation between 3 and 10 days according to the species’ growth conditions.

After this incubation time, 300 mg of wet sample was transferred to cryotubes containing 0.25 ml of acid-washed glass beads (Sigma-Aldrich, Diegem, Belgium) put at −80 °C during 40 min and freeze-dried overnight with a freeze-dryer Epsilon 1-6D (Martin Christ, Osterode am Harz, Germany). Freeze-dried fungi were subsequently beat-beaten with a Mini bead beater (Biospec Products, OK, USA) during 1 min at maximal speed.

The total DNA was extracted with an adapted phenol chloroform (24:1) protocol (Ashktorab and Cohen 1992) and purified with the Qiagen CTAB genomic Tip-20 kit (Qiagen Benelux, B.V., KJ Venlo, the Netherlands) according to the manufacturer’s recommendation. DNA was eluted with 100 μl Gibco® DNase, RNase, protease free water (Life Technologies, Gent, Belgium). The DNA integrity was verified on a 2 % agarose gel. The DNA amount and purity were evaluated with a Nanodrop® 2000 (Thermo Scientific, Wilmington, USA).

### Design of primers

All the at the time of primer design in NCBI GenBank (https://www.ncbi.nlm.nih.gov/genbank/) available 18S rDNA sequences from *A. versicolor* strains as well as from other closely related species (namely *A. creber*, *A. fumigatus*, *A. sydowii*, *P. chrysogenum*) were collected and aligned with the “MegAlign” software V10.0.1 (Lasergene, Madison, USA) to identify the sequence region of interest. The publicly available sequences of 18S rDNA used were for *A. versicolor*, AJ937751.1/AJ937753.1/AJ937754.1/AJ937755.1/AM883155.1/AM883156.1/AY728196.1/EF125026.1/EU042148.1/FJ878627.1/FJ878625.1/FJ461692.1/FJ904814.1/KJ466864.1/JN205048.1, for *A. creber*, KJ775474.1, for *A. fumigatus*, KC411924.1/KC237295.1/KC237291.1/KC237292.1/KC142152.1/HE864321.1/KC119199.1/KC119200.1/JX944178.1/JX944118.1, for *A. sydowii*, DQ114468.1/FJ807779.1/HQ625522.1/JN94914.1/KJ775568.1/KJ775569.1/KJ775570.1/KJ775571.1/KJ775574.1, and for *P. chrysogenum*, JN903544.1/JN798499.1/JX535315.1/AF033465.1/HQ336383.1/GU325676.1/EU709771.1/JX996985.1/JF834167.1/FJ004280.1.

Then, different primer pairs were designed in the regions of ITS 1 and ITS 2 with the “Primer 3 V.0.4” software (http://bioinfo.ut.ee/primer3-0.4.0/) (Untergasser et al. 2007). Primer dimers and secondary structure formation were evaluated during the design with Primer 3. An in silico specificity test was performed with the “wprimersearch” software (https://wemboss.uio.no/wEMBOSS/) (Sarachu and Colet 2005). This in silico PCR simulation allows selecting the primer pairs that only amplify the targeted sequences. The specificity of the primers was also verified using BLASTn (http://blast.ncbi.nlm.nih.gov/Blast.cgi). In total, seven primer pairs have been designed (Table 2).

**Table 2:** Primer sequences developed in silico

Name	Purpose	Sequence 5′ to 3′
***Aversi_ITS_*** **r**	**Reverse primer**	**AGTTCGCTGCGTTCTTCATC**
*Aversi_ITS_*r2	Reverse primer	CTGCATCACTCTCAGGCATG
*Aversi_ITS_*f1	Forward primer	CTGAGAGTGATGCAGTCTGAG
*Aversi_ITS_*f2	Forward primer	CCCACCCGTGACTACCTAA
***Aversi_ITS_*** **f**	**Forward primer**	**CTGAGAGTGATGCAGTCTGAGTCTG**
*Aversi_ITS_*f2	Forward primer	TGCCTGAGAGTGATGCAGTCTGAGTCTGA
*Aversi_ITS_*f3	Forward primer	CTGAGAGTGATGCAGTCTGAGTCAG
*Aversi_ITS_*f4	Forward primer	GAGTGATGCAGTCTGAGTCTG
*Aversi_ITS_*f5	Forward primer	CTTCATGCCTGAGAGTGATGCAGTCTGC

Primers in bold selected as the most specific for the selected region of *A. versicolor*, yielding an amplicon of 53 base pairs

### Qualitative SYBR® green qPCR assay

The qPCR assay (*Aversi_ITS* qPCR method) was performed with the SYBR® green chemistry using a real-time PCR IQ5™ system from Bio-Rad (Temse, Belgium).

The standard reaction mix (25-μl final volume) contained 12.5 μl of 2× SYBR® green PCR Mastermix (Diagenode, Liège, Belgium), 0.25 μl of *Aversi_ITS* forward and reverse primers (0.2 μM), and 7 μl of Gibco® DNase, RNase, protease free water (Life Technologies, Gent, Belgium). To this mix, 5 μl of genomic DNA (gDNA) at 200 theoretical genomic copy numbers per microliter was added. The number of genomic DNA copies was calculated according to the formula presented below:

$$ {C}_{\eta }=\frac{m\times {A}_c}{M_w\times {G}_s} $$

with *C*_*n*_ as genomic copy number, *m* as the amount of gDNA (grams) and determined by Nanodrop® 2000 (Thermo Scientific, Wilmington, USA), *Ac* as the Avogadro’s constant (Mohr et al. 2008), *M*_*w*_ as base pair mean molecular weight (649 Da), and *G*_*s*_ as genome size (expressed in basepairs) of *A. versicolor* = 33,130,000 bp (Joint Genome Institute 2014). There is only publicly available information on the size of the genome of one specific *A. versicolor* strain. This size was taken to calculate the number of genomic DNA copies for all *A. versicolor* strains, although some strain-dependent deviations may exist.

All the runs were performed using following thermal cycling conditions: 1 cycle of 95 °C for 10 min (Taq activation), followed by 40 amplification cycles of 15 s at 95 °C (denaturing step) and the annealing and extension step at 60 °C for 1 min. Afterwards, a melting curve was performed with a gradual increase of temperature of 0.5 °C/6 s from 55 to 95 °C during 15 min. The threshold level for the reaction was automatically determined by the Bio-Rad IQ 5 software V. 2 (Bio-Rad, Temse, Belgium).

In each reaction, a “no template control” (NTC) was included for the analysis, i.e., the DNA template was replaced by ultrapure water in the reaction mix. This NTC allowed verifying that no contamination occurred and that no primer dimers were formed.

### Strain confirmation: sequencing and theoretical *T*_m_ calculation

In order to confirm their identity, the ITS 1 and 2 regions of *A. versicolor* (IHEM 18884, IHEM 1323, IHEM 1355, IHEM 2023, IHEM 2157, IHEM 2788, IHEM 6598, IHEM 9674, IHEM 10351, IHEM 19014, IHEM 19210, IHEM 19256, IHEM 29832, IHEM 22014, IHEM 2757, IHEM 24424), *A. creber* (IHEM 2646), and *A. sydowii* (IHEM 895, IHEM 1360, IHEM 20347) were verified by Sanger sequencing analysis on an ABI3130xl Genetic Analyzer apparatus (Applied Biosystems, Life Technologies, Gent, Belgium) with the BigDye Terminator v3.1 cycle sequencing kit (Applied Biosystems, Life Technologies, Gent, Belgium) according to the manufacturer’s recommendations. The ITS 1 and 2 regions were firstly amplified with the primers ITS1F (5′-CTTGGTCATTTAGAGGAAGTAA-3′) (Gardes and Bruns 1993) and ITS4 (5′-TCCTCCGCTTATTGATATGC-3′) (White et al. 1990) and then sequenced with the primers ITS1 (5′-TCCGTAGGTGAACCTGCGG-3′) and ITS2 (5′-GCTGCGTTCTTCATCGATGC-3′) (White et al. 1990). The consensus sequence of each of the targeted regions was made based on the forward and reverse sequence. These sequences were aligned with the MEGA v6.06 (http://www.megasoftware.net/) software and visualized with the CLC sequence viewer v7.0.2 (Qiagen Benelux, B.V., KJ Venlo, the Netherlands). The consensus sequences were also compared to the sequences available in the NCBI database by using BLASTn (http://blast.ncbi.nlm.nih.gov/) in order to confirm the amplification of the targeted DNA regions and their identity.

Based on these sequences, the theoretical *T*_m_ of the amplicon obtained in the *Aversi_ITS* assay was calculated in silico with the online tool OligoAnalyzer 3.1 from IDT (http://eu.idtdna.com/calc/analyzer) (IDT, Leuven, Belgium) under the PCR conditions described above (White et al. 1990).

### *Aversi_ITS* assay: performance assessment

#### Selectivity test

The selectivity test was composed of two steps as previously reported for the validation of qPCR methods for food pathogens (Barbau-Piednoir et al. 2013a, b). Firstly, a preliminary selectivity test was carried out on the target species (*A. versicolor* IHEM 18884) and two nontarget species (i.e., *A. fumigatus* IHEM 3562 and *P. chrysogenum* IHEM 20859), using 15,000 theoretical copies of gDNA.

Secondly, a larger selectivity test was performed, evaluating the inclusivity (the selected primers should amplify DNA of each tested strain from the tested target species) and the exclusivity (DNA of nontarget species close to the target or described to frequently occur in the same environment with the target species should not be amplified by the selected primers) of the *Aversi_ITS* qPCR method.

The experimental design of the full selectivity test was adapted from Barbau-Piednoir et al. (2013a), i.e., 16 target strains (*A. versicolor*) and 14 nontarget strains were included, i.e., 4 from the *Aspergillus* section *Versicolores* (Jurjevic et al. 2012) (*A. creber* IHEM 2646, *A. sydowii* IHEM 895, IHEM 1360, IHEM 20347) and 10 of the most common indoor airborne fungi (Beguin and Nolard 1994) (Table 1).

Each qPCR was performed with the SYBR® green technology under the conditions described above using a total of 1000 theoretical copies of gDNA per reaction (evaluated for each target with its own corresponding genome size).

#### Dynamic range and efficiency estimation

The linearity of this SYBR® green qPCR assay was assessed based on the qPCR analysis of a serial dilution, in duplicate, of gDNA (1000, 500, 100, 50, 10, 5, 2, and 1 theoretical copy number of gDNA) obtained by two independent extractions of *A. versicolor* IHEM 18884. This analysis gives the possibility to assess two parameters, i.e., the coefficient of determination (*R*^2^) and the PCR efficiency. *R*^2^ is an indicator of the correlation of the data regarding the linear regression curve. The PCR efficiency (*E*) calculation was previously described in Rutledge and Cote (2003). According to the most recent guidelines developed for GMO detection with qPCR SYBR® green (ENGL 2015), the *R*^2^ and amplification efficiency are not applicable to qualitative methods. However, a *R*^2^ ≥ 0.98 and a PCR efficiency ranging between 80 and 120 % have previously been indicated as performance criteria for the validation of qualitative qPCR methods (Broeders et al. 2014).

#### Sensitivity test: limit of detection

To evaluate the sensitivity of the *Aversi_ITS* assay, a serial dilution of gDNA of *A. versicolor* IHEM 18884 was performed to determine the limit of detection (LOD). The LOD is defined as the lowest concentration of an analyte which is detected with a probability of 95 % (Barbau-Piednoir et al. 2013b).

To estimate this LOD, seven dilutions of gDNA of *A. versicolor* IHEM 18884 (i.e., 10, 5, 2, 1, 0.5, 0.2, and 0.1 theoretical copies of gDNA) were tested in six independent runs, each with six repetitions. The qPCR conditions applied here were the same as those described above. The LOD should be below 25 copies according to definition of minimum performance requirements for analytical methods of GMO testing (ENGL 2015).

#### Repeatability calculation

As previously described (Barbau-Piednoir et al. 2013b), the repeatability limit (*r*) of the *Aversi*_*ITS* qPCR method was evaluated with the same experimental design than that applied for the LOD evaluation (see section “Sensitivity test: limit of detection”). This *r* value is defined as the maximal difference of two results obtained under identical experimental conditions with a probability of 95 % (Barbau-Piednoir et al. 2013b).

The relative standard deviation of the repeatability (*RSDr*) was calculated as the absolute value of the coefficient variation and expressed in percentage. For these criteria, there is no limit fixed for qualitative qPCR methods (ENGL 2015). The *RSDr*, evaluated for the *C*_q_ values, should be ≤25 % for all dilutions above the LOD for quantitative methods (ENGL 2015).

#### Environmental testing: inhibition test

Before qPCR analysis of the environmental samples, an inhibition test was performed in order to verify that no inhibition from the collection liquid, used for air sampling with the Coriolis *µ* air sampler (Bertin Technology, Montigny-le-Bretonneux, France), occurred during the qPCR. To this end, 300 mg of an *A. versicolor* IHEM 18884 culture was spiked, in duplicate, into 15 ml of collection liquid, i.e., ultrapure water containing Tween® 20 0.01 % (Sigma-Aldrich, St Louis, USA). Then, the spiked solutions were centrifuged at 5000*g* during 15 min. The supernatant was removed, the pellet was suspended in 1.5 ml of Gibco® DNase, RNase, protease free water (Life Technologies, Gent, Belgium), and the DNA was extracted according to the protocol described above. Afterward, each undiluted DNA extract and a 10-fold dilution thereof were analyzed with the *Aversi*_ITS qPCR assay. Theoretically, the *C*_q_ difference (∆*C*_q_) between the *C*_q_ value of the 10-fold diluted and the undiluted sample is 3.3 when the PCR efficiency is 100 %. The experimental ∆*C*_q_ is calculated based on the obtained *C*_q_ for both samples (difference between the obtained *C*_q_ for the 10-fold diluted and the undiluted sample). Taking into account the PCR efficiency of each run, we considered that there are no PCR inhibitors in the DNA extract if the experimental ∆*C*_q_ value for the 10-fold dilution equals 3.3 ± 0.5.

#### Environmental testing: proof of concept

To assess the performance of the *Aversi_ITS assay* to detect *A. versicolor* in real-life samples, two houses contaminated by fungi were studied in the framework of the activities of the CRIPI (Cellule Régionale d’Intervention en Pollution Intérieure from Brussels Environment, Brussels, Belgium). In each house, two sets of air samples were independently collected for classical and molecular analysis respectively, in minimum four different rooms, i.e., the bedroom, bathroom, living, and kitchen. In each procedure, air samples were taken at the height of a seated person. To avoid any contamination by air flow, each room was as much as possible isolated from outside by closing windows and doors.

A first set of samples of air (0.08 m^3^ for each sample) were collected and analyzed according to the procedure previously defined (Nolard et al. 2004) and used in routine by the CRIPI (Nolard et al. 2004). Briefly, air contaminants were directly sampled on Sabouraud chloramphenicol agar (Bio-Rad, Temse, Belgium) slipped on HYCON® Agar Strips (Merck, Darmstadt, Germany), using the RCS plus (Biotest, Rupperswil, Switzerland) air sampler following the manufacturer’s instructions and with a flow rate of 80 l/min during 1 min.

After an incubation of 5 days at 25 °C for mesophilic and 2 days at 45 °C for thermophilic fungi, the species determination was performed by microscopic visualization (Nolard et al. 2004). The level of contamination was evaluated by counting of the colony-forming units (CFU) on plate and expressed as CFU/m^3^ according to the guidelines defined by Nolard and coworkers (2004).

The second set of air samples (1.5 m^3^ for each sample) were taken in duplicate with the Coriolis *µ* air sampler (Bertin Technology, Montigny-le-Bretonneux, France) and collected in 15 ml of ultrapure water containing Tween® 20 (0.01 %) (Sigma-Aldrich, St Louis, USA) in an appropriate sterile cone (Bertin Technology, Montigny-le-Bretonneux, France). A flow rate of 300 l/min during 5 min was applied in each room. In each room, two cones were collected; the first one was analyzed by culturing and microscopic visualization, the second one by qPCR for the detection of *A. versicolor.* The first cone was put at −80 °C until analysis.

To analyze the Coriolis *µ* air samples with the culturing approach, the samples were centrifuged at 5000*g* during 15 min, and the supernatant was discarded. The pellet was suspended in 1.5 ml of Gibco® DNase, RNase, protease free water (Life Technologies, Gent, Belgium) and plated on Sabouraud chloramphenicol agar (Bio-Rad, Temse, Belgium), using an inclusion method. Hereto, under laminar flow, the 1.5-ml samples were put into empty petri dishes. Afterward, Sabouraud chloramphenicol medium was poured into the plates and kept at room temperature until the medium was solidified. The plates were incubated 5 days at 25 °C for mesophilic and 2 days at 45 °C for thermophilic fungi as it was done for the routine protocol described above. The species determination was performed by microscopic visualization (Nolard et al. 2004).

To analyze the Coriolis *µ* air samples by qPCR for the presence of *A. versicolor*, samples were first centrifuged at 5000*g* during 15 min, and the supernatant was discarded. Next, the pellet was suspended in 1.5 ml of Gibco® DNase, RNase, protease free water (Life Technologies, Gent, Belgium), and the extraction was performed with the DNA extraction protocol used for pure cultures (see section “Culture conditions and DNA extraction”). The DNA amount and purity were evaluated with a Nanodrop® 2000 (Thermo Scientific, Wilmington, USA). The qPCR reactions were performed on 10 μl of eluted DNA, i.e., 10 % of the total DNA eluted from in 1.5 m^3^ of air sampled, corresponding to the amount of DNA mentioned in Table 5.

## Results

### Design and selection of *A. versicolor* qPCR primer pair

**Fig. 1:**
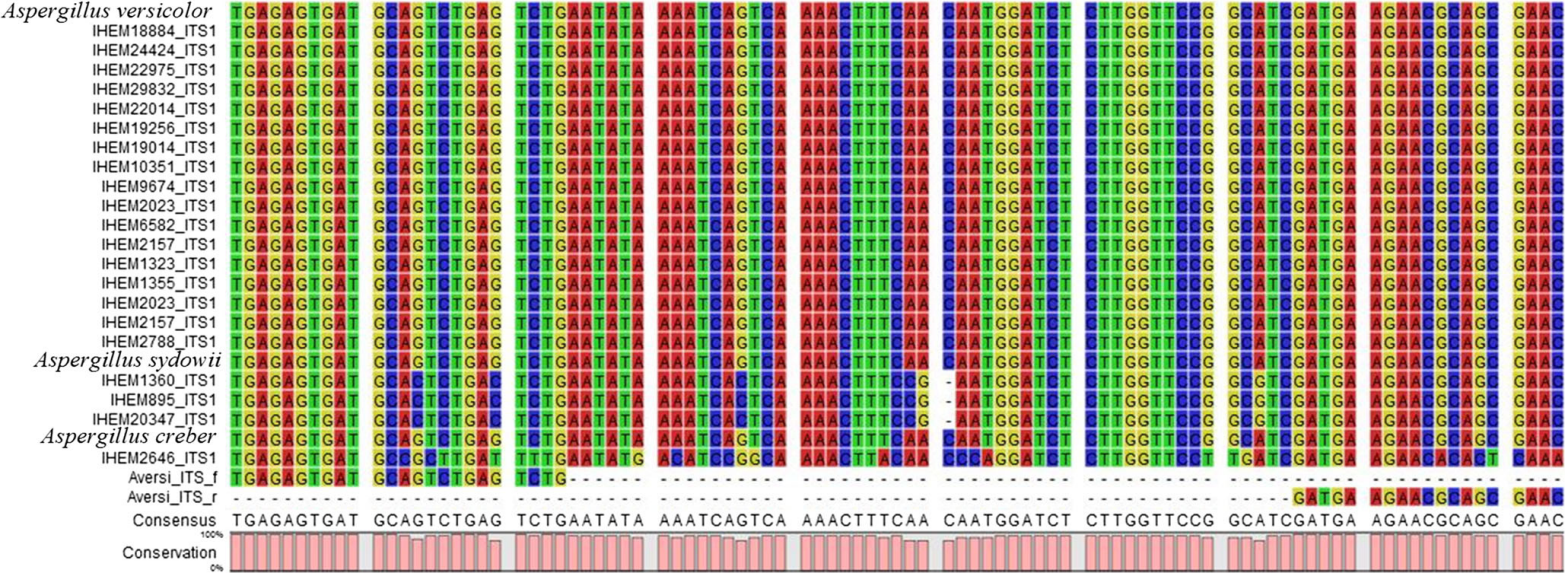
Alignment of selected forward and reverse *Aversi*_*ITS* primers on ITS1 region sequences of *A.* *versicolor*, *A. creber*, and *A. sydowii*. This alignment was made using publicly available ITS sequences of *Aspergilus versicolor*, *A. creber*, and *A. sydowii*, extended with ITS sequences from the strains from the BCCM/IHEM collection used during the validation of the qPCR assay (indicated with IHEM prefix) and with the primers designed in this study (*Aversi_ITS*_f and *Aversi_ITS*_r). Because no nucleotide variation was detected for each of the public sequences used, only one sequence was introduced for this alignment, as a representative for that species. The accession numbers of all the NCBI sequences used in this study are listed hereunder, i.e., for *A.* *versicolor* AJ937751.1/AJ937753.1/AJ937754.1/AJ937755.1/AM883155.1/AM883156.1/AY728196.1/EF125026.1/EU042148.1/FJ878627.1/FJ878625.1/FJ461692.1/FJ904814.1/KJ466864.1/JN205048.1, for *A. creber* KJ775474.1, for *A. sydowii* DQ114468.1/FJ807779.1/HQ625522.1/JN94914.1/KJ775568.1/KJ775569.1/KJ775570.1/KJ775571.1/KJ775574.1. The ITS1 region of the BCCM/IHEM strains of *A. versicolor* (16), *A. creber* (1) and *A. sydowii* (3) used during the performance assessment of the qPCR assay was sequenced and aligned to those publicly available. Consensus (last line of the alignment) corresponds to a consensus sequence defined by the software. The conservation level among each sequence (0 to 100 % of conservation) is represented by the pink rectangles at the bottom of the figure

To select the *A. versicolor* primers, all the at the time of the primer design publicly available *A. versicolor* 18S rDNA sequences were searched. This collection of sequences was extended with those available for *A. creber* and *A. sydowii*. These species are both members of the *Versicolores* group as is also *A. versicolor*. These three species are difficult to be morphologically distinguished. Based on an in silico analysis of these sequences, seven forward primers and two reverse primers targeting a conserved region of the full ITS 1 region of *A. versicolor* were designed (Table 2, Fig. 1).

Unfortunately, in the conserved ITS1 region for *A. versicolor*, it was not possible to design specific primers which are not conserved in *A. creber* and *A. sydowii* (Fig. 1). The amplicons obtained with the *Aversi_ITS* f/r primers present 100 % of identity when using the sequences for these three species available in NCBI (Fig. 1). Preliminary experimental specificity tests were performed on DNA of *A. versicolor*, *A. fumigatus*, and *P. chrysogenum*. The *Aversi_ITS*_1f/r primer pair (Fig. 1, Table 2) was selected for the detection of *A. versicolor* as it was the unique combination of forward and reverse primers allowing the amplification of *A. versicolor* DNA and not that of the closely genetically related *A. fumigatus* and *P. chrysogenum* (data not shown).

#### *Aversi_ITS* assay: performance assessment

The performance of this *Aversi_ITS* assay was assessed according to the guidelines defined for the validation of qPCR detection and identification methods in other fields (Barbau-Piednoir et al. 2013b; Broeders et al. 2014; ENGL 2015). The performance assessment includes the evaluation of the following criteria: the selectivity of the primers, the dynamic range, the PCR efficiency, the LOD, and the *Aversi_ITS* assay repeatability.

#### Selectivity of the qPCR SYBR® green *Aversi_ITS* assay

**Fig. 2:**
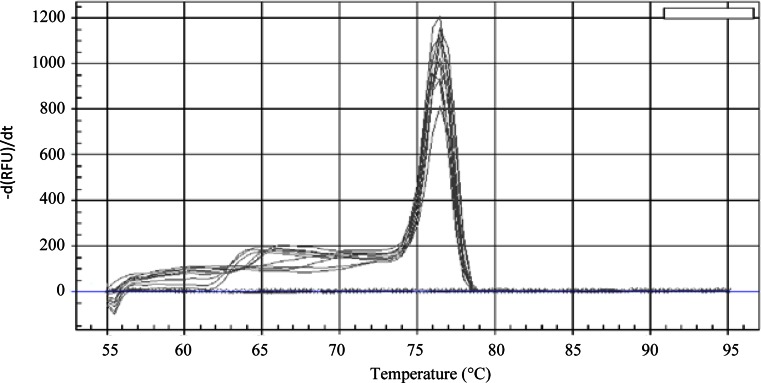
Melting curves obtained with the *Avesi_ITS* qPCR assay for the *A. versicolor* pure strains listed in Table 1. The melt curves were obtained with the Bio-Rad IQ 5 software V. 2 (Bio-Rad, Temse, Belgium). The *x* axis shows the temperature (°C). The *y*-axis presents the inverse of the first derivative of the best-fitted curve of the measured fluorescence decrease. The *grey curves* correspond to the *A. versicolor* listed in Table 1. The *blue flat curves* represent the nontemplate controls

The assessment of the selectivity was performed in two steps, i.e., an inclusivity and an exclusivity test. To perform the inclusivity test, DNA extracted from 16 BCCM/IHEM strains of *A. versicolor* was amplified. DNA of all the strains was amplified (16/16) with a *C*_q_ range of 24.26 ± 0.44 to 28.67 ± 0.23 for 1000 copies of gDNA. The *T*_m_ values ranged between 76.25 ± 0.35 and 76.75 ± 0.29 °C (Table 1, Fig. 2). The sequence of these BCCM/IHEM strains matches perfectly the ones published on NCBI for the corresponding region. All of the amplicons showed 100 % identity (Fig. 1). The obtained *T*_m_ for each amplicon corresponds to the theoretical *T*_m_ (76.50 °C) (Fig. 2).

Subsequently, the exclusivity test was performed using DNA of nontarget species (*A. creber*, *A. sydowii*, *A. fumigatus*, *A. alternata*, *C. cladosporoïdes*, *C. herbarum*, *C. sphaerospermum*, *P. chrysogenum*, *S. charatum*, *U. botrytis*), closely related to *A. versicolor* and/or occurring in the same environment, i.e., mold species from indoor air. As expected based on the in silico analysis, no DNA from nontargeted species considered as a background set of indoor air fungal species (i.e., *A. fumigatus*, *A. alternata*, *C. cladosporoïdes*, *C. herbarum*, *C. sphaerospermum*, *P. chrysogenum*, *S. charatum*, *U. botrytis*) was amplified during the test (Table 1), except for the *A. sydowii* (three strains tested IHEM 895, 1360, 20347) and *A. creber* (IHEM 2646) strain. Although the in silico analysis of the targeted ITS region of *A. versicolor*, *A. creber*, and *A. sydowii* with the available sequences in the NCBI database did not show any sequence dissimilarities (Fig. 1), for a same genome copy number (i.e., 1000 theoretical genomic copies), the *C*_q_ values ranged between 30.18 ± 0.13 and 37.34 ± 1.84 for *A. sydowii* and 30.70 ± 0.70 for *A. creber* (Table 1). To clarify this issue, the obtained amplicons were sequenced. The sequence of the ITS1 amplicon obtained for the experimentally tested *A. creber* (IHEM 2646) differs from that of *A. versicolor* with 17 nucleotides, of which seven in the annealing site of the primers. Amplicons of *A. sydowii* IHEM 895, IHEM 1360, IHEM 20347 differ with eight nucleotides of which three in the annealing site of the forward primer (Fig. 1). But despite of the differences in nucleotide composition, the obtained *T*_m_ values for the amplicon of *A. creber* and *A. sydowii* were in the same range as that of the target *T*_m_ (76.25–76.75) with respectively 76.50 and 76.25 °C (Table 1). The BLAST analysis of the ITS1 and ITS2 region obtained by sequencing confirmed the IHEM 895, IHEM 1360, and IHEM 20347 as *A. sydowii* with 93 % of identity and the IHEM 2646 as *A. creber* with 81 % of identity. Despite this latter low identity, the strain was confirmed as a *A. creber* based on morphological analysis and sequencing of the regions 5.8 S and ITS2 by BCCM/IHEM (data not shown).

In each assay, an NTC was included to verify that no contamination occurred during the preparation of the qPCR mixes and filling of the 96-well plates. In none of the assays, a signal was observed for the NTC. This absence of signal also demonstrates that no dimerization of primers occurred during the analysis, as predicted during the in silico test (Fig. 2).

#### Limit of detection and PCR repeatability

Based on 6 independent runs with a total of 18 repetitions (Table 3), the LOD for this SYBR® green assay was determined as being one or two copies of *A. versicolor* genomes (*C*_q_ 35.32 ± 0.78 and 34.65 ± 0.91) (Table 4). The *r* and *RSDr* were respectively 2.4 and 6.889 % for the *C*_q_ value at LOD.

**Table 3:**
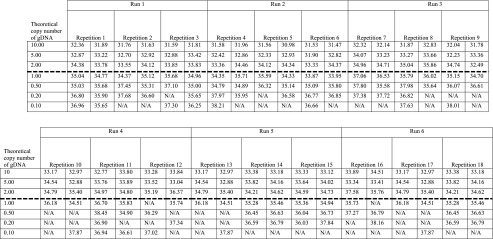
*C*_q_ values obtained during the six runs of the limit of detection estimation for qPCR SYBR® green assay *Aversi_ITS*

Mean of C_q_ value obtained for six repetitions (repetitions 1 to 18) of six independent runs (runs 1 to 6) of a serial dilution of genomic DNA of *A. versicolor* (concentration expressed in copy number of genomes of the IHEM 18884 strain). The LOD is defined by the dashed line

**Table 4:**
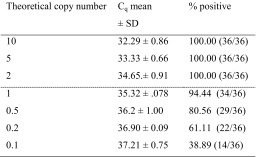
Limit of detection results (*C*_q_ mean, SD and % positive) for *Aversi_ITS* SYBR® green qPCR assay

Mean of *C*_q_ value obtained for six repetitions of six independent runs of a serial dilution of genomic DNA of *A. versicolor* (concentration expressed in theoretical copy numbers of genomes of the IHEM 18884 strain), the standard deviation (±SD) and the percentage of positive response observed at each dilution point. The number of positive signal per assay is given between brackets. The LOD is defined by the dashed line

#### Dynamic range and PCR efficiency

**Fig. 3:**
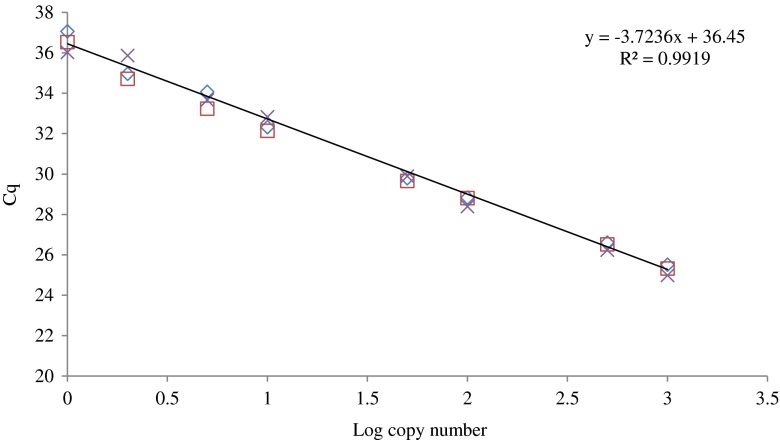
*R* ^*2*^ and PCR efficiency of the *Aversi_ITS* qPCR assay. The PCR efficiency (E) was evaluated in duplicate on a serial dilution of gDNA (1000 to 1 theoretical copy number of gDNA) obtained by two independent extractions of *A. versicolor* IHEM 18884. The coefficient of determination (*R* ^*2*^) regarding a linear correlation curve. Log copy number/logarithm of the theoretical copy number of gDNA. *C*_q_/*C*_q_ values obtained by qPCR for each repetition of each gDNA dilution

Based on a dilution series of 8 levels, corresponding to 1000 to 1 theoretical genomic copies of *A. versicolor*, the dynamic range and efficiency of the *Aversi_ITS* assay were determined. Between the tested range, a linear model with a *R*^2^ of 0.9919 and an efficiency of 88.34 % were obtained for this qPCR method (Fig. 3).

#### Proof of concept: environmental testing

At first, an inhibition test was performed to verify that no inhibition occurred during the amplification of the DNA extracted from the environmental samples. Theoretically, with a 100 % efficient amplification, a 10-fold dilution corresponds to a *C*_q_ difference of 3.3. The obtained *C*_q_ value was 25.06 ± 0.34 for the undiluted DNA extract of the collection fluid spiked with pure *A. versicolor* and 28.28 ± 0.64 for the 10-fold dilution. This test showed that no inhibition of the amplification occurs.

The *Aversi_ITS* assay was subsequently tested on environmental samples collected with the Coriolis *µ*-air sampler and compared to the results obtained with classical detection and identification method used by CRIPI in routine. Additionally, environmental samples collected with the Coriolis *µ* were also analyzed using culturing and microscopic visualization, in order to be able to evaluate whether possible observed differences are due to the differences in the sampling method or in the detection method used (Table 5).

**Table 5:** Environmental sampling, comparison of classical analysis methods with the SYBR® green qPCR *Aversi_ITS* assay

Classical method			Coriolis *μ* sampler and culturing	Molecular method			
RCS plus sampler and culturing				Coriolis *μ* sampler and qPCR			
Sampling place	Species	Number of colonies per plate	CFU/m^3^ ^a^	Number of colonies per plate	Amount of DNA/PCR reaction (ng)^b^	*C*_q_ mean ± SD^c^	Theoretical copy number of gDNA for 1 m^3^ ^d^
House 1							
Room	*A. versicolor*	3	38	9	19.8	32.15 ± 0.49	67
	*P. chrysogenum*	7	88	17			
	Infertile mycelium	0	0	1			
Kitchen	*A. versicolor*	1	13	2	21.7	35.25 ± 0.21	7
	*A. glaucus*	1	13	1			
	*P. chrysogenum*	8	100	7			
	Yeast (undetermined)	1	13	1			
Living room	*P. chrysogenum*	24	300	25	53.3	N/A	/
Bathroom	*A. versicolor*	4	50	6	50	30.26 ± 0.35	93
	Infertile mycelium	4	50	4			
	*P. chrysogenum*	15	188	17			
House 2							
Room 1	Infertile mycelium	4	50	4	51.5	N/A	/
	*P. chrysogenum*	17	213	15			
Room 2	Infertile mycelium	2	25	1	10.4	N/A	/
	*P. chrysogenum*	3	38	4			
Kitchen	*P. chrysogenum*	6	75	5	12.5	N/A	/
	Infertile mycelium	3	38	4			
Living room	*A. versicolor*	1	13	2	49.5	35.85 ± 0.07	7
	*P. chrysogenum*	18	225	20			
Bathroom	*A. versicolor*	1	13	2	45	33.9 ± 0.28	33
	*Cladosporium spp.*	1	13	1			
	*P. chrysogenum*	15	188	13			
	Yeast (undetermined)	0	0	2			

*N/A* no amplification, i.e., *A. versicolor* was considered as not detected in the sample

^a^The value for CFU/m^3^ is an estimation of fungal contamination based on the number of colonies per plate. The Coriolis *µ* samples that were put into culture to serve as a qualitative control for the species identified with the RCS plus sampler and for *A. versicolor* detected in the Coriolis samples with the qPCR method

^b^Ten microliters of extracted DNA from 1.5 m^3^ sampled air (and eluted in 100 μl of water) were used in a 25-μl -PCR reaction

^c^ *C*_q_ values are *C*_q_ means (≤40) ± standard deviation (SD) obtained with the validated *Aversi_ITS* primers

^d^Theoretical copy number of gDNA based on the *Aspergillus versicolor* IHEM 18884 strain defined as the strain of reference for the validation of the *Aversi_ITS* assay (Table 4)

With the classical routine method (culturing, counting and microscopic visualization), *A. versicolor* was detected in the two sampled houses with a quantity ranging between 13 and 50 CFU/m^3^. *P. chrysogenum* was found as the most important contaminant in each of the sampled houses with a range of 75 to 300 CFU/m^3^. Other taxa, i.e., *Aspergillus glaucus*, *Cladosporium* spp., and an undetermined species, were also observed, though in the same range of quantities as *A. versicolor*. These taxa occur in the list of most frequently found fungal contaminants of indoor environments. Infertile mycelia were also present in the sampled houses. The range of counts for *A. versicolor* correlated with the results obtained with the *Aversi_ITS* qPCR assay. For each sample where *A. versicolor* was identified by microscopic analysis, a positive qPCR signal ranging between *C*_q_ 30.26 ± 0.49 and *C*_q_ 35.85 ± 0.07 was obtained (Table 5). The samples where *A. versicolor* was not detected on plate gave a negative signal in qPCR.

Similar observations were made for the results obtained with culturing of the Coriolis *µ* air samples. For the different sampling places, the same species as identified for the samples collected with the RCS plus sampler were found, within two rooms (room of house 1 and the bathroom of house 2) some additional undetermined species detected for the Coriolis *µ* sampler. The number of colonies on plate for these species was for both air samplers in the same range, including for *A. versicolor*. As for the RCS plus-based sampling, also for the air samples collected with the Coriolis *µ*, for each sample where *A. versicolor* was identified by microscopic analysis, a positive qPCR signal was obtained (Table 5). A negative signal in qPCR was obtained for the samples where *A. versicolor* was not detected on plate.

## Discussion

Currently, indoor fungal contamination is considered as an important public health problem (World Health Organization 2009). Among the different species described, *A. versicolor* is considered as one of the most important (Beguin and Nolard 1994; Benndorf et al. 2008; de Ana et al. 2006; Melkin et al. 2004) and is suspected to have a link with asthma (Mendell et al. 2011; Sharpe et al. 2014; Verhoeff and Burge 1997). However, to provide more scientific-based, causal evidence on this, efficient screening and monitoring of indoor airborne fungal communities are crucial and necessary.

In this study, we developed a SYBR® green qPCR method for the detection of *A. versicolor*. This *Aversi_ITS* assay targets the ITS1 region, which is the same sublocus than the one targeted by the TaqMan®-based EPA assays (including the one for *A. versicolor*) (Haugland et al. 2004). This region from the 18S rDNA is considered as the most variable locus of this complex, and therefore, it is the most efficient species marker for fungi (Nilsson et al. 2008), making it the most appropriate region of the fungal DNA for the development of a molecular screening tool. This some intraspecies variability of ITS sequence might impact TaqMan®-based methods and the probe’s hybridization when different strains of a specific species need to be detected with the same probe. An alternative to the hydrolysis probe can be found in the SYBR® green chemistry. Less expensive than TaqMan-based assays, SYBR® green assays theoretically allow for species specificity and species discrimination based on a melting curve and the *T*_m_ value of the amplicon.

Most published PCR and qPCR assays to detect molds are not uniformly assessed for their performance with clear guidelines or norms (Costa et al. 2001; Haugland et al. 2004; Johnson et al. 2012; Melkin et al. 2004; Roussel et al. 2013). In the present study, we propose a strategy to evaluate the performance of qPCR assays applied to mold detection. Therefore, using the same strategy than the one published for foodborne pathogens (Barbau-Piednoir et al. 2013b), the guidance existing for GMO was used to select a set of performance criteria for the qPCR assay (Broeders et al. 2014; ENGL 2015); namely, the selectivity, PCR efficiency, dynamic range, sensitivity, and repeatability parameters were assessed to evaluate the performance of our developed *Aversi_ITS* assay.

First, the results of the specificity test showed that all of the tested *A. versicolor* strains were detected with the *Aversi_ITS* assay. However, for an identical number of genomic copies (i.e., 1000 gDNA copy numbers), a variation of approximately 4 *C*_q_ between these strains was observed. Because no differences in the ITS sequences were observed between the tested BCCM/IHEM stains (Fig. 1), including the sites of primer annealing, this difference in *C*_q_ might be explained by variation in the copy number of the 18S rDNA operon to which the targeted ITS region belongs. This ITS region is known to vary not only between species but also within the species (Black et al. 2013; Corradi et al. 2007; Iwen et al. 2002; Schoch et al. 2012). For example, for *A. fumigatus*, the intraspecies variation factor was estimated at 2.5, implying that the 18S rDNA gene complex could vary between 38 and 91 copies per genome (Herrera et al. 2009). A similar variation rate for *A. versicolor* might explain the observed difference in *C*_q_ values between different strains of this species. However, this hypothesis needs more investigation of the copy number of 18S rDNA in *A. versicolor* strains, similar to what was described by Herrera et al. for *A. fumigatus* (Herrera et al. 2009). Consequently, qPCR assays developed on the ITS region, such as this *Aversi_ITS* assay, are qualitative detection methods. To develop a quantitative method based on ITS, the copy number of the 18S rDNA should be determined for each target strain of the species.

The *Aversi_ITS* assay did not amplify DNA extracted from 10 nontarget strains selected among the most common airborne fungal species. This indicates a good selectivity of the *Aversi_ITS* assay (Beguin and Nolard 1994). However, the assay did amplify DNA of two other members of the *Aspergillus* section *Versicolores*, i.e., *A. creber* and *A. sydowii*. This was expected, based on the primer sequences and the alignments of already publicly available ITS sequences, which were identical to the one of *A. versicolor* in the selected region. These species are very close and their ITS sequences in general differ only in a few nucleotides (Hinrikson et al. 2005; Jurjevic et al. 2012). However, the *C*_q_ obtained for the amplicons of *A. sydowii* and *A. creber* were not in the expected range based on the identity of the publicly available sequences, although they resulted in the expected *T*_m_. Therefore, additional tests for strain identification were performed. These additional tests confirmed the identity of the strains used for the exclusivity test. They also confirmed the obtained experimental *T*_m_, coinciding with the one calculated based on the obtained sequence which however shows mismatches as compared to the ITS1 target region of *A. versicolor* or of the publicly available sequences of *A. creber* and *A. sydowii* used for the primer design. Although normally the *T*_m_ allows for species discrimination, in the selected amplicon region this is not the case for *A. versicolor*, *A. creber*, and *A. sydowii*. The obtained *C*_q_ is however higher for *A. creber* and *A. sydowii* for the same amount of template DNA, which might be due to the mismatches in the primer annealing sites, in addition to the difference in ITS copy number. These observations demonstrate the need for more publicly available sequences for these closely related species to be used for qPCR assay development.

These nontarget amplifications of *A. sydowii* were also previously reported for the TaqMan® assays of EPA with probes *Avers2* and *Asydo3* developed respectively for the detection of *A. versicolor* and *A. sydowii* (Haugland et al. 2004; United States Environmental Protection Agency 2014). These probes amplified each time both species. *A. creber* is a recently described species, isolated from indoor air samples and identified through multilocus DNA sequencing (Jurjevic et al. 2012), and has not yet been commonly used for the testing of TaqMan® *A. versicolor* specific assays. However, these aspecific detections should have a limited impact on a possible use of the *Aversi_ITS* assay in routine analysis in Belgium. Indeed, *A. creber* and *A. sydowii* are not frequently detected in indoor environment, as compared to *A. versicolor.* Further investigations are needed to evaluate the difference between these three species concerning the impact of their presence in indoor air on public health.

To improve the discrimination between these three closely related species based on merely molecular methods, an alternative approach should be developed. One possibility could be the use of a combination of different markers similar to what has already been done for the detection of GMO (Van den Bulcke et al. 2010) and foodborne pathogens (Barbau-Piednoir et al. 2015). The presence or the absence of an amplification signal for each of the markers and their combination defines a decision-taking tree. For fungi, this approach to improve the species discrimination could be possible if we would combine our primers of the *Aversi_ITS* assay with primers targeting another gene marker or a discriminatory SNP. In case that the sample is composed of a mixture of DNA from different species (e.g., from an air sample), the results will hint to a “candidate mold” which should be further confirmed by downstream analysis, such as sequencing. This idea is supported by Schoch and coworkers in 2012 who suggested that the use of a combination of different DNA regions, like ITS 1 and *β*-tubulin or another region, i.e., the DNA barcode principle, should improve the phylogenetic analysis of fungi (Schoch et al. 2012). Hereto, more genomic sequences of both species (*A. creber* and *A. sydowii*) should become publicly available in order to design primers for such discriminatory regions.

Subsequently, the PCR linearity and efficiency were evaluated as quality criteria for the developed assay. The BCCM/IHEM 18884 strain was collected and purified from a contaminated house by CRIPI and is used as a reference strain for allergy studies by the CRIPI. Therefore, this strain was selected as a reference to determine the parameters of our qPCR assay during the performance assessment.

The efficiency of this assay (*E*) was estimated to be 88.34 %, which complies with the guidelines for qualitative qPCR methods (Broeders et al. 2014). Our method is also characterized by a high *R*^2^ value (0.9919) which demonstrated the linearity of our assay and of the accuracy of our experimental setup.

Moreover, the *Aversi_ITS* assay is sensitive with a LOD defined as one or two genomic copies per reaction, and therefore, it complies with the acceptance criteria, i.e., LOD below 25 copies (ENGL 2015). This LOD is similar to the results reported by Johnson et al. (2012) for their qPCR detection method of *A. fumigatus* which has an LOD ranging between 6 and 0.6 genomes (Johnson et al. 2012). Our *Aversi_ITS* showed also to be repeatable, with a *RSD*_*r,*_ for all dilutions above the LOD, lower than 25 %, thereby fulfilling the requirements of the validation guidelines used in this study.

As discussed above, we mainly used the guidelines for GMO detection for the evaluation of our qPCR assay. As it was recently shown (Barbau-Piednoir et al. 2013a), these guidelines were successfully used to evaluate with high standard the performance of the SYBR® green qPCR methods for the detection of bacterial pathogens. However, requirements defined for food and feed analysis are not necessarily adapted for indoor airborne fungi monitoring, as the laws and the control measures are more numerous for food than for microbiological air pollution and the impact of food contamination on public health is more documented and better understood than for fungal air contamination. The increasing development and use of molecular tools for the identification and the monitoring of indoor airborne microbiological contamination imposes the establishment of guidelines for harmonization of performance requirements and threshold values for the parameters of the qPCR assays. Ultimately, this may contribute to the establishment of standardized and reproducible microbiological methods, highly needed to determine a causal link between indoor fungal contamination and health effects.

To deliver a proof-of-concept of our developed qPCR assay aimed to be used for indoor fungal contamination monitoring, the *Aversi_ITS* assay was tested on environmental samples and results were compared to those obtained with traditional methods of screening (i.e., culture, CFU counting and microscopic identification). This last part of our study confirmed that the *Aversi_ITS* assay is a valuable alternative for the currently used classical methods. The results of the qPCR assay were comparable to the ones obtained with the classical routine protocol as to indicate whether the targeted species *A. versicolor* was present or not, and this using a shorter time period to obtain these results with the qPCR method, i.e., 2 days maximum for the qPCR analysis (including DNA extraction with an overnight lyophilization step and qPCR analysis) compared to 5 to 10 days for the classical methods. Based on the obtained *C*_q_ and an extrapolation from the *A. versicolor* data obtained for the LOD estimation, a theoretical copy number of *A. versicolor* gDNA per cubic meters of air could be estimated in order to attempt a more detailed comparison of the results from molecular and the classical methods (Table 5). The theoretical number of genomic copies for *A. versicolor* ranged between 7 and 67 for 1 m^3^ of sampled air for a number of CFU/m^3^ ranging between 13 and 50 for those collected with the RCS plus air sampler in the routine classical method. A similar range of colonies of *A. versicolor* was obtained for the samples collected with the Coriolis *µ*. The culturing of Coriolis *µ* samples was included as a control to verify that different samplers do not lead to different species detected on plate, especially for *A. versicolor.*

The *A. versicolor* amounts obtained are in a similar range when comparing the culturing results to the qPCR results, despite the following considerations. The small difference in amounts could be attributed to the sampling method, the performance of the sampler used, and the conversion factor used to express the results in m^3^, which are different for the RCS plus and Coriolis *µ* sampling methods. Also, the possibility that one colony would grow from an aggregate of fungal cells should be taken into account, explaining why the results are not exactly coinciding. Additionally, qPCR will also include the nonculturable *A. versicolor* fraction, while this fraction remains undetected using the classical methods. It is also important to note that the estimation of gDNA copies was based on the strain IHEM 18884, which is not necessarily the strain that was present in the contaminated houses. As observed during the evaluation of the assay, the real copy number of the ITS regions of the strains detected here could differ from that of the strain IHEM 18884. This would lead to another amount of gDNA copies. Nevertheless, the trend for the contamination by *A. versicolor* was similar as detected by the different methods, i.e., the house 1 was the most contaminated and bathrooms presented more contamination than other rooms.

Our environmental test also showed, as expected, that other species than *A. versicolor* are present inside buildings. Therefore, it will be interesting to develop and assess a more exhaustive multiplex tool targeting different indoor airborne fungal species. Different types of multiplex technologies can be envisaged, including the SYBR® green technology, where the discrimination between species could be based on the *T*_m_ values of the amplicons obtained with universal primers for the ITS region. However, this might imply the use of more accurate technologies such as high-resolution melting (HRM) qPCR to perform the discrimination of some closely related taxa. Indeed, some species differ by a few nucleotides and are hardly discriminated with traditional qPCR methods. Based on a very fine temperature gradient, HRM could be an interesting tool to perform the discrimination and the identification of the airborne fungal community (McCarthy et al. 2013). It would also be interesting to investigate whether HRM in combination with the primers of the *Aversi_ITS* assay might offer a solution to discriminate the three species of the *Aspergillus* section *Versicolores.*

In conclusion, the developed *Aversi_ITS* method based on the SYBR® green chemistry is a convenient qualitative assay for the detection of *A. versicolor.* With the increased risk for public health linked to the augmentation of indoor fungal contamination, such a molecular assay is a first step in offering a valuable alternative to the currently used classical methods in the framework of routine monitoring of indoor air fungal contamination by a public health laboratory. A reduced time of detection of both the culturable and nonculturable fungal fraction allows to reduce the time of reaction and taking of measurements aiming at reducing the impact on human health. With an extension toward other important indoor airborne fungal contaminants, tools as the one developed in this study, based on harmonized guidelines to be urgently established, will contribute to an improved indoor air quality monitoring and ultimately to an improved insight into the causal effect between indoor airborne fungal contaminants and health effects. Once this causal link is established, meaningful regulatory individual exposure standards for well-defined airborne mold allergens can be established (AIHA 2011; Verhoeff and Burge 1997).
